# Perceptions of Telemental Health Care Delivery During COVID-19: A Cross-Sectional Study With Providers, February-March 2021

**DOI:** 10.3389/fpsyt.2022.855138

**Published:** 2022-04-04

**Authors:** Hattie Wilczewski, Samantha R. Paige, Triton Ong, Janelle F. Barrera, Hiral Soni, Brandon M. Welch, Brian E. Bunnell

**Affiliations:** ^1^Doxy.me Research, Doxy.me Inc., Rochester, NY, United States; ^2^Department of Psychiatry and Behavioral Neurosciences, University of South Florida, Tampa, FL, United States; ^3^Biomedical Informatics Center, Medical University of South Carolina, Charleston, SC, United States

**Keywords:** telemedicine, telemental health, mental health, quality of care, COVID-19

## Abstract

The COVID-19 pandemic accelerated adoption of telemental health (TMH). Providers with limited TMH experience faced challenges during the rapid switch to remote patient care. We investigated TMH providers’ perceptions about remote care one year into the pandemic according to *when* providers adopted telemedicine (i.e., before *vs.* after March 2020) and *how much* of their caseloads were served remotely (i.e., < 50% *vs.* ≥ 50%). Between February–March 2021, 472 TMH providers completed a cross-sectional, web-based survey that measured perceived benefits and satisfaction with telemedicine, therapeutic alliance, patient-centered communication, eHealth literacy, multicultural counseling self-efficacy, and facilitating factors of using telemedicine. Providers who began using telemedicine before the pandemic reported having better training, task-related therapeutic alliance with patients, and ability to conduct multicultural interventions, assessments, and session management. Providers who served ≥ 50% of their caseload remotely reported greater satisfaction with their practice, stronger beliefs about the benefits of telemedicine, and greater perceived effects of telemedicine on alleviating the impact of COVID-19. There were no differences in reports of patient-centered communication nor eHealth literacy. In conclusion, providers who adopted TMH more recently may require additional training and support to successfully establish a working alliance with their patients, especially with multicultural aspects of care.

## Introduction

Coronavirus Disease 2019 (COVID-19) ignited a shift in mental health care from in-person to remote delivery. In response to the pandemic, studies estimate over 97% of mental health providers have adopted telemental health (TMH) to supplement or replace in-person care (1, 2). Some mental health providers’ caseloads increased by 25–50% during the pandemic with patient surges as high as 6,558% (1, 3). Telemedicine revolutionized the delivery of evidence-based mental health care (4), and proved to be a dependable solution that 90% of providers surveyed intend to use beyond the resolution of COVID-19 (5). It is imperative to understand how mental health providers deliver remote services to inform and sustain post-pandemic TMH care models.

Mental health providers were highly satisfied with telemedicine before the pandemic, despite its slow uptake (2, 6). High satisfaction and benefits of TMH have been attributed to its convenience, the ability to reach more patients, and the opportunity for a better work-life balance (7). The COVID-19 pandemic led to an abrupt transition from in-person care to TMH for most providers, making TMH less of a choice and more of a requirement to continue practicing (8). In a study conducted during the initial months of the pandemic, mental health providers practicing in the state of Florida believed they were still delivering high-quality care and communicating effectively with their patients despite the transition to TMH care (2). We are now years into the pandemic and it is unknown whether TMH providers remain satisfied with their capacity to conduct high-quality care. The purpose of this study is therefore to further explore perceptions of delivering high-quality healthcare remotely among a nationally representative United States sample of TMH providers.

High-quality healthcare is effective, safe, and equitable and delivered by a provider who clearly communicates and involves patients in health decisions (9). Therapeutic alliance–the relationship and tasks to achieve mutually established health goals–is reliably among the strongest predictors of mental health treatment success, making strategies and tactics for building a strong patient-provider relationship paramount to high-quality care (10–14). Consistent with high-quality care, therapeutic alliance thrives in patient-centered environments where providers elicit the “true” wishes of patients to recognize and respond to their needs and values (15). For in-person healthcare settings, patient-centered communication is commonly described as asking and welcoming questions to understand patients’ beliefs and needs to ensure that healthcare is concordant with their values (16). The capacity to practice patient-centered communication requires providers to practice cultural competencies, or multicultural counseling self-efficacy (17). Patient-centeredness is integral to the capacity of providers to recognize and become responsive to the diverse backgrounds of patients and integrate their values into clinical decision-making (18). In TMH settings, patient-centered communication occurs when providers help patients navigate the telemedicine platform, which includes facilitating an environment where they can access, evaluate, and discuss online resources as partners in care (i.e., eHealth literacy) (19). Therapeutic alliance, eHealth literacy, and multicultural competence in a patient-centered environment are vital for success in mental health care, but it is unclear how these indicators of high-quality care have fared throughout the pandemic.

Delivering high-quality TMH care is also attributed to organizational factors that facilitate or support providers in using telemedicine. A recent study found that having strong organizational capabilities, such as sufficient information technology infrastructure, is integral to successful telemedicine adoption in healthcare systems (20). However, healthcare providers must also feel supported in using telemedicine to practice their specialty with fidelity and to effectively provide care to their patients (21). This includes feeling confident that the overarching healthcare system is supportive of telemedicine utilization (e.g., timely reimbursement processes), as well as having enough training or resources available to help them most effectively practice their specialty remotely. Harst et al. (22) found that perceptions of organizational factors which impact telemedicine use has most often been explored among patients rather than providers. This is a significant gap in the literature that our study aims to address.

Telemental health providers’ perceptions about the quality of care they provide to patients remotely may vary according to when they adopted telemedicine and how frequently they use it. For example, Zhu et al. (1) found that TMH providers were more comfortable with telemedicine during the pandemic than before it began. Other than this finding, little empirical attention has been paid to examining how the temporal aspects of TMH uptake affect perceptions of TMH care delivery. The decision whether to use telemedicine largely depends on the healthcare provider, making them gatekeepers of telemedicine (23, 24). Therefore, it is imperative to understand how perceptions of TMH care vary according to when it was adopted and the frequency of its use.

The purpose of this study was to investigate TMH providers’ perceptions about TMH care delivery during the pandemic. Participants were surveyed about their perceptions of TMH satisfaction, benefits, therapeutic alliance, patient-centered communication, eHealth literacy, cultural competence, and organizational factors that facilitate TMH use. A secondary purpose of the study was to examine how perceptions of TMH care vary depending on when telemedicine was adopted (before or after the onset of COVID-19) and the proportion of caseload served remotely (<50% or ≥50%). Our investigation occurred in Spring 2021, approximately one year after global leaders announced the COVID-19 pandemic.

## Materials and Methods

### Sample and Procedures

Telemental health providers (*N* = 472) completed a cross-sectional, web-based survey between February and March 2021. Emails were sent to TMH providers who used the Doxy.me telemedicine platform, sampling from which has shown to be consistent with mental health industry demographics (1, 2, 7, 25, 26). After providing electronic informed consent, providers completed a series of screening questions. English-speaking adults (i.e., ≥ 18 years) who identified as practicing mental and/or behavioral health providers were eligible to participate. Providers were compensated with a free 1-month Doxy.me professional membership. Study procedures were approved by the Institutional Review Board of the University of South Florida (IRB#002053).

### Survey and Measures

The survey was iteratively developed and refined based on prior studies exploring TMH practice (1, 2, 7). The survey included a variety of items selected from validated scales, questions adapted from validated scales, and novel questions related to TMH practice during COVID-19. See Table 1 for Cronbach’s alpha reliabilities for each measure.

**TABLE 1:** Perceptions of care by telemedicine adoption and caseload.

Construct and Measure	Telemedicine Adoption		Telemedicine Caseload	
	<March 2020 M(*SD*)	≥March 2020 M(*SD*)	<50% M(*SD*)	≥50% M(*SD*)
**Benefits of Telemedicine**				
Satisfaction with Practice (0.86)	4.19 (0.79)	4.15 (0.68)	3.93 (0.86)	4.21 (0.66)**
Protects Against COVID-19 (0.81)	4.49 (0.68)	4.51 (0.70)	4.13 (0.99)	4.59 (0.57)***
Improves Practice (0.70)	3.13 (1.17)	3.02 (1.06)	2.50 (1.08)	3.18 (1.06)***
**Therapeutic Alliance**				
Goals (0.78)	4.07 (0.65)	3.99 (0.66)	3.92 (0.71)	4.03 (0.64)
Tasks (0.81)	4.22 (0.52)**	4.05 (0.60)	4.07 (0.63)	4.11 (0.57)
Bonds (0.81)	4.49 (0.48)	4.41 (0.50)	4.44 (0.44)	4.44 (0.51)
**Patient-Centered Communication**				
Encourage Open Communication (0.88)	4.19 (0.85)	4.09 (0.85)	3.97 (0.88)	4.16 (0.83)^†^
Confidence in Providers’ Ability (0.88)	4.34 (0.90)	4.23 (0.78)	4.15 (0.83)	4.29 (0.82)
Support Patients After/Outside of the Session (0.91)	4.06 (0.91)	3.97 (0.82)	3.88 (0.90)	4.03 (0.84)
Improve Comfort with the Technology (0.67)	3.69 (0.94)	3.59 (0.80)	3.47 (0.88)	3.65 (0.83)^†^
**eHealth Literacy**				
Information Awareness (0.83)	3.88 (0.84)	3.75 (0.83)	3.73 (0.88)	3.81 (0.82)
Information Seeking (0.81)	3.96 (0.79)	3.84 (0.83)	3.83 (0.82)	3.88 (0.81)
Information Evaluation (0.84)	4.04 (0.90)	3.90 (0.88)	3.93 (0.84)	3.95 (0.90)
**Multicultural Counseling Self-Efficacy**				
Intervention (0.91)	3.98 (0.61)*	3.85 (0.54)	3.85 (0.56)	3.90 (0.57)
Assessment (0.86)	3.39 (0.86)**	3.11 (0.77)	3.31 (0.75)	3.17 (0.82)
Session Management (0.90)	4.07 (0.55)*	3.92 (0.53)	3.97 (0.52)	3.96 (0.55)
**Facilitating Factors**				
Feeling supported to practice *via* telemedicine (0.78)	4.00 (0.67)	4.13 (0.76)	4.25 (0.71)*	4.05 (0.75)

Cronbach’s alpha reliabilities are in parentheses next to the construct or measure name. *p < 0.05; **p < 0.01; ***p < 0.001; ^†^p < 0.10.

#### Personal and Professional Characteristics of Telemental Health Providers

We collected demographic (e.g., age, gender, race, ethnicity, rurality) and professional characteristics (e.g., professional title, theoretical orientation, disorders treated, age group primarily treated, change in overhead costs).

#### Beliefs About the Satisfaction and Benefits of Telemedicine Experience

Perception of providers’ satisfaction with telemedicine experience was measured using several items reported in Slone et al. (2). These items were linearly rescaled to create a unidimensional satisfaction measure. Each item was anchored on a 5-point Likert scale from *Extremely Dissatisfied* to *Extremely Satisfied*. Benefits of telemedicine (general) was measured using 3 items anchored on a scale from 1 = *Not at all* to 5 = *Extremely*. Benefits of telemedicine specific to COVID-19 was similarly measured using 3 items anchored on a scale from 1 = *Not at all* to 5 = *Extremely*.

#### Therapeutic Alliance

Therapeutic alliance with patients *via* telemedicine was captured using the Working Alliance Inventory - Short Revised – Therapist version (WAI-SR-T) (27). This measure consists of three subscales: goals, tasks, and bonds. Responses ranged from 1 = *Seldom* to 5 = *Always*.

#### Patient-Centered Communication

Patient-centered communication *via* telemedicine was measured with an 11-item instrument. Based on best practices in patient-centered communication (28, 29), we identified four subscales: encourage expression, increase confidence in ability, support patients outside the session, and help patients overcome technology issues. Responses ranged from 1 = *Very difficult* to 5 = *Very easy*.

#### Electronic Health Literacy

Electronic health (eHealth) literacy was measured based off items from the eHealth Literacy Scale (eHEALS) (30) adapted to fit the therapist perspective. For example, “I know what health resources are available on the internet” was rephrased as “I know what health resources are available on the Internet for my clients.” We identified three subscales adapted from a prior eHEALS 3-factor model study (31): information awareness, information seeking, and information evaluation. Responses were anchored on a 5-point Likert scale from 1 = *Strongly disagree* to 5 = *Strongly agree*.

#### Multicultural Counseling Self-Efficacy

Multicultural counseling competence was measured with the Multicultural Counseling Self-Efficacy Scale – Racial Diversity Form (MCSE-RD) (17). The measure focuses on MH providers’ confidence in multicultural counseling skills with racially diverse clients, a central aspect of multicultural competence. We measured three subscales: multicultural intervention, multicultural assessment, and multicultural counseling session management. The 60 items in the MCSE-RD were reduced to 22 by consulting two clinical content experts. Criteria for inclusion included eliminating redundancies in scale items and item relevance to TMH practice. Each subscale displayed adequate internal reliability. Responses were anchored on a 5-point Likert scale from 1 = *No confidence at all* to 5 = *Complete confidence*.

#### Facilitating Factors

Finally, we measured the degree that providers receive organizational support to use telemedicine (e.g., training, resources) using a 4-item measure with response ranging from 1 = *Strongly disagree* to 5 = *Strongly agree*.

### Data Analysis

SPSS v28 (IBM Corp.) was used for all analyses. Descriptive and frequency statistics were computed to describe the sample and responses to survey items. A series of χ^2^ tests were conducted to examine how demographic factors varied according to our two independent variables (IVs), which included when providers began using telemedicine (0 = before March 2020; 1 = March 2020 or later) and how much of their caseload was served remotely (0 ≤ 50%; 1 ≥ 50%). A series of independent samples *t*-tests were also conducted to examine how perceptions of care quality varied by both IVs. Statistical significance was set at *p* < 0.05.

## Results

Table 2 shows that TMH providers in this study were, on average, 53.19 years old (*SD* = 13.16) and predominantly female (81.36%), white (80.51%), and non-Hispanic (91.10%). Most (72.68%) providers lived in a Metropolitan area, with either a moderate or strong urban influence.

**TABLE 2:** Personal characteristics of TMH providers (*N* = 472).

Personal Characteristics	*n*	(%)
**Age (years), *M (SD)***	53.19	13.16
**Sex**		
Female	384	81.36
Male	79	16.74
Other	3	0.64
Missing	6	1.27
**Race**		
White	380	80.51
Black or African American	30	6.36
American Indian/Alaska Native	6	1.27
Asian	8	1.69
Native Hawaiian or Pacific Islander	2	0.42
Multiracial	20	4.24
Other	18	3.81
Missing	8	1.69
**Ethnicity**		
Hispanic, Latinx, or Spanish origin	37	7.84
Not Hispanic/Latino	430	91.10
Missing	5	1.06

Table 3 shows the professional characteristics of the providers. Most identified as mental health counselors (47.46%), psychologists (31.14%), and social workers (14.19%). Nearly three-quarters of providers (75.42%) reported working in an individual practice and 18.43% in a network of providers or a small clinic. Over half of providers primarily treated anxiety and mood related disorders (i.e., anxiety, 43.01%; mood, 21.82%; trauma- and stressor-related disorders, 24.79%), followed the cognitive-behavioral treatment paradigm (54.24%), and served adults (18-64 years old; 83.90%). Private health insurance was the most common form of reimbursement for telemedicine services. About half (45.34%) of providers said their overhead costs (including rent, supplies) had not changed because of providing telehealth services. Over half (67.58%) of providers (*n* = 319) started using telemedicine March 2020 or later, and 79.66% (*n* = 376) reported seeing at least 50% of their patients *via* telemedicine. There was no statistically significant association between percent of caseload served *via* telemedicine and whether telemedicine was adopted before or during the pandemic (*p* = 0.20).

**TABLE 3:** Professional characteristics of TMH providers (*N* = 472).

Professional Characteristics	*n*	(%)
**Professional Title**		
Mental Health Counselor	224	47.46
Psychologist	147	31.14
Social Worker	67	14.19
Marriage and Family Therapist	33	6.99
Missing	1	0.21
**Type of Mental Health Practice**		
Individual Practice	356	75.42
Small clinic or network of providers	87	18.43
Health organization (i.e., hospital, large clinic, gov’t agency)	15	3.18
Educational setting (i.e., school, college, university)	5	1.06
Missing	9	1.91
**Primary Age Group Treated**		
Children (0-10 yrs old)	13	2.75
Adolescents (11-17 yrs old)	47	9.96
Adults (18-64 yrs old)	396	83.90
Older adults (65 + yrs old)	7	1.48
Missing	9	1.91
**Most Common Mental Health Disorder Treated**		
Anxiety	203	43.01
Mood	103	21.82
Trauma- and stressor-related	117	24.79
Other	40	8.47
Missing	9	1.91
**Primary Treatment Paradigm**		
Behavioral	11	2.33
Cognitive-Behavioral	256	54.24
Existential/Humanistic	46	9.75
Family Systems	24	5.08
Interpersonal	61	12.92
Psychodynamic/analytic	61	12.92
Social Learning	4	0.85
Missing	9	1.91
**Geographical Region**		
Metropolitan/City (Urban center)	110	23.31
Strong urban influence	68	14.41
Moderate urban influence	165	34.96
Weak urban influence	62	13.14
Rural/Small Town (Remote - no urban influence)	62	13.14
Missing	5	1.06
**Primary Health Insurance Reimbursement**		
Public Insurance (Medicare, Medicaid)	68	14.41
Private Insurance	312	66.10
Client out-of-pocket	83	17.58
Other	9	1.91
**Change in Overhead Costs**		
Greatly decreased	63	13.35
Decreased some	120	25.42
No change	214	45.34
Increased some	56	11.86
Greatly increased	10	2.12
Missing	9	1.91

### Personal and Professional Characteristics of Telemental Health Providers

Most providers who used telemedicine, regardless of onset, were women; however, the proportion of women who used telemedicine was significantly greater during the COVID-19 pandemic (85.48%) than before it (77.46%), χ^2^ (1, *N* = 459) = 4.48, *p* < 0.05. No other statistically significant relations existed for personal demographics.

Providers who used telemedicine to treat the majority (50% or more) of their caseload were more likely to treat adults (18 + years old) rather than children and adolescents (0-17 years old), χ^2^ (1, *N* = 463) = 11.10, *p* < 0.05. There were no statistically significant differences in changes to overhead costs because of adopting telemedicine technology. However, providers who served less than 50% of their caseload reported that overhead costs “haven’t changed” (*M* = 2.91; *SD* = 0.60) whereas providers who served more than 50% of their caseload *via* telehealth reported that overhead costs have “decreased some” (*M* = 2.57; *SD* = 0.99), *t* (461) = 3.07, *p* < 0.01. This difference should be noted as having a moderate effect (Cohen’s *d* = 0.42). No other statistically significant relationships existed for professional characteristics and telemedicine use.

### Beliefs About the Satisfaction and Benefits of Telemental Health Care

Table 1 includes the responses to general satisfaction of using telemedicine and the benefits (i.e., general to telemedicine and specific to protecting against COVID-19 transmission). Providers reported feeling somewhat satisfied with their TMH practice (*M* = 4.16; *SD* = 0.71). They believed that telemedicine services moderately benefitted their practice (*M* = 3.05; *SD* = 1.09) but that it had been very-to-extremely beneficial in protecting against the spread of COVID-19 while supporting continuity of care (*M* = 4.50; *SD* = 0.69). The timing of telemedicine adoption (before or after March 2020) was not associated with providers’ satisfaction using telemedicine or its perceived benefits. However, compared with their counterparts who served fewer patients remotely, providers who served 50% or more of their caseload remotely reported greater satisfaction with their telemedicine practice (*M* = 4.22 *SD* = 0.66 vs. *M* = 3.93 *SD* = 0.86), *t* (371) = −3.14; 95% CI = −0.47, −0.11; *p* < 0.01. Providers who served most of their caseload remotely also reported stronger beliefs about the benefits of telemedicine to support their practice (*M* = 3.18 *SD* = 1.06 vs. *M* = 2.50 *SD* = 1.08), *t* (421) = −5.50; 95% CI = −0.94, −0.42; *p* < 0.001). They were also more likely to report that telemedicine helped to alleviate the impacts of COVID-19 (*M* = 4.59 *SD* = 0.57 vs. *M* = 4.13 *SD* = 0.99), t (421) = −5.13; 95% CI = −0.62, −0.20; *p* < 0.001.

### Therapeutic Alliance

Table 1 shows that providers reported very often agreeing with their patients on the therapeutic goals (*M* = 4.01; *SD* = 0.65) and tasks to achieve those goals (*M* = 4.10; *SD* = 0.58) *via* telemedicine. Providers also felt they very often-to-always established a meaningful bond with the patients they served remotely (*M* = 4.44; *SD* = 0.50). Compared with providers who started using telemedicine during the pandemic, providers who used telemedicine before the pandemic reported having a greater task-related alliance with their patients (*M* = 4.22 *SD* = 0.52 vs. *M* = 4.05 *SD* = 0.60), *t* (357) = 2.62; 95% CI = 0.04, 0.31; *p* < 0.01. There were no other statistically significant differences in therapeutic alliance sub-scores based on when providers began using telemedicine or the proportion of caseload served remotely.

### Patient-Centered Communication

Table 1 also shows that providers generally felt that it was somewhat easy to encourage patients to openly communicate *via* telemedicine (*M* = 4.12; *SD* = 0.85), to increase patients’ confidence in their ability as a healthcare professional (*M* = 4.26; *SD* = 0.82), and to stay engaged with them outside the telemedicine session (*M* = 4.00; *SD* = 0.85). They reported it was “neither easy nor difficult” to help patients feel more comfortable using telemedicine (*M* = 3.62; *SD* = 0.84). There were no statistically significant differences in patient-centered communication based on when providers began using telemedicine or how much of their caseload is served remotely. However, two subscales approached statistical significance based on the percent of patients seen *via* telemedicine; providers who served more than 50% of their caseload remotely felt it was easier to encourage their patients to openly communicate (*p* = 0.06) and help them feel more comfortable to use telemedicine (*p* = 0.07).

### eHealth Literacy

In Table 1, providers somewhat agreed that they were knowledgeable about where to find health information on the Internet to benefit their patients (*M* = 3.79; *SD* = 0.83), how to help their patients find health information on the Internet (*M* = 3.87; *SD* = 0.81), and how to help their patients evaluate the quality of health information they find on the Internet (*M* = 3.94; *SD* = 0.89). There were no statistically significant differences in online health information awareness, seeking, and evaluation skills according to when providers began using telemedicine and the percentage of caseload they served remotely.

### Multicultural Counseling Self-Efficacy

Table 1 includes the responses to providers’ multicultural counseling self-efficacy. Providers reported some confidence in their ability to conduct multicultural assessment (*M* = 3.19; *SD* = 0.81) and some-to-a lot of confidence in their ability to conduct multicultural interventions (*M* = 3.89; *SD* = 0.56) and multicultural counseling session management (*M* = 3.96; *SD* = 0.54). Compared to providers who began using telemedicine March 2020 or later, providers who used telemedicine before the COVID-19 pandemic reported a statistically significant higher ability to conduct: (a) multicultural interventions (*M* = 3.98 *SD* = 0.61 vs. *M* = 3.85 *SD* = 0.54), *t* (344) = 2.00; 95% CI = 0.00, 0.26; *p* < 0.05, (b) multicultural assessments (*M* = 3.39 *SD* = 0.86 vs. *M* = 3.11 *SD* = 0.77), *t* (344) = 3.07; 95% CI = 0.10, 0.47; *p* < 0.01, and (c) multicultural counseling session management (*M* = 4.07 *SD* = 0.55 vs. *M* = 3.92 *SD* = 0.53), *t* (344) = 2.35; 95% CI = 0.02, 0.27; *p* < 0.05. There was no statistically significant difference in multicultural counseling self-efficacy based on the percentage of their caseload served remotely.

### Facilitating Factors

Table 1 shows that providers somewhat agreed they were adequately trained and supported to provide services *via* telemedicine (*M* = 4.11; *SD* = 0.74). This perception was stronger among providers who began using telemedicine before rather than during the COVID-19 pandemic (*M* = 4.25 *SD* = 0.71 vs. *M* = 4.05 *SD* = 0.75), *t* (409) = 2.54; 95% CI = 0.05, 0.36; *p* < 0.05. Perceptions about facilitating factors did not vary according to percentage of patients served remotely.

## Discussion

The current study aimed to investigate TMH providers’ perceptions about remote healthcare delivery one year into the pandemic. A secondary aim was to examine the variability in these perceptions according to when TMH providers adopted telemedicine (i.e., before or during the pandemic) and how much of their caseload was served remotely (i.e., less than 50%; 50% or more). Approximately 80% of providers in this study, regardless of whether they adopted telemedicine before or during the pandemic, reported treating at least half of their patient caseload *via* telemedicine. Findings demonstrate heterogeneity in TMH providers’ perceptions of delivering care *via* telemedicine.

### Principal Results

Telemental health providers generally reported being satisfied with using telemedicine to deliver care one year into the COVID-19 pandemic. Providers believed that telemedicine was beneficial to their practice and to the safety of themselves and their patients during the COVID-19 pandemic. Positive beliefs were consistent among providers who adopted telemedicine before or during pandemic. However, they were strongest among providers who used telemedicine to treat 50% or more of their caseload. In previous research, TMH providers have cited telemedicine as a convenient and considerably low-cost approach to reach patients who otherwise would not have access to care (7). Although TMH providers were generally satisfied with their telemedicine experience, positive beliefs about using telemedicine to deliver care were cultivated when the technology was regularly integrated into their practice.

TMH providers generally felt confident in their ability to establish a therapeutic alliance with their patients. This is a positive finding, as a therapeutic alliance is an integral component of effective mental health care (14). TMH providers reported establishing treatment goals with their patients, despite the challenges of cultivating task-related alliances. Specifically, TMH providers who began using telemedicine during the pandemic reported the weakest task-oriented alliances with their patients. There are several barriers that may impede the ability of providers to achieve mutual understanding and agreement on exercises to help their patients achieve treatment goals. Some examples include poor internet connection, challenges using devices and software, limited knowledge about how to engage patients remotely, and believing that patients are unreceptive to telemedicine (32). In a study conducted prior to the pandemic (26), TMH providers commonly assigned patients exercises that involved coping and emotional regulation, problem solving, mindfulness, interpersonal skills, and modifying and addressing core beliefs. Future research is needed to examine if and how these exercises are conducted by mental health providers who began using telemedicine during the pandemic. Such inquiry would be useful to inform instructional efforts to help providers new to telemedicine to succeed in cultivating therapeutic alliances.

The strongest therapeutic alliances are cultivated within patient-centered environments, meaning that care is discussed and coordinated with the patients’ needs, preferences, and values in mind (18). Telemedicine can challenge the patient-centeredness and therapeutic alliances of healthcare appointments, as self-expression and relational connections among other considerations may manifest differently than in-person appointments (33, 34). As a result, telemedicine has a reputation for being provider-centered, as observational analyses of clinical encounters have found that providers exhibit verbal and information dominance (35, 36). And although there is enthusiasm for telemedicine as a patient-centered healthcare delivery solution (37), a study conducted in the early phases of the pandemic found that disparities in patient-centered communication exist *via* telemedicine (e.g., limited opportunities for open-ended communication and poorly expressed empathy) (38). Future research is needed to capture both patient and provider assessments of therapeutic alliance following telehealth appointments.

There are two important findings related to patient-centered care and communication in this study. First, TMH providers believed it was somewhat easy to encourage their patients to openly communicate about their feelings, values, and needs *via* telemedicine. This positive perception about facilitating open communication was consistent regardless of when providers began using telemedicine and how frequently it is used to serve their caseload. Second, providers also felt it was somewhat easy to help patients feel confident in their abilities as a remote healthcare professional. Patients are more likely to ask for providers to repeat information *via* telemedicine than in-person consultations (35). As a result, providers may perceive patients’ expressions of perceptual difficulty as engagement, giving them greater opportunity to exhibit their knowledge about subject matter. Future research examining remote patient-centered communication and investigating its effect on how care is delivered by providers and received by patients is a fruitful area.

Another aspect of patient-centered communication is helping patients feel comfortable receiving and navigating health care. In this study, TMH providers in this study felt it was neither easy nor difficult to help their patients feel comfortable receiving care *via* telemedicine. Further, they felt somewhat knowledgeable about where to find online health information and how to help their patients evaluate its quality to support their health-related goals. Nearly 60% of healthcare providers have shared and recommended online health information to their patients (39), and this proportion is expected to be higher now that the internet has penetrated the daily lives of most people worldwide. Future research is needed to explore what online health information is discussed during telemedicine appointments. Understanding what content is introduced during these appointments and exploring the process by which the information is shared and navigated will inform future interventions to support providers in this endeavor.

To appropriately establish patient-centered care and cultivate therapeutic alliance among racially/ethnically diverse patients, TMH providers must be capable of providing culturally sensitive treatments. TMH providers reported some confidence in their ability to apply multicultural competencies in mental health assessment, intervention, and session management. Multicultural counseling self-efficacy was strongest among providers who reported using telemedicine before the pandemic. This difference may be due to differences in the amount of experience using telemedicine to deliver culturally sensitive care, or perhaps the availability of cultural competence training. Despite a great deal of heterogeneity in workforce cultural competence trainings, common strategies include increasing providers’ knowledge and skills to facilitate culturally competent care (40). Future research might focus on patients demographics and include observational studies of multicultural counseling competencies in practice *via* telemedicine. Overall, findings of this study echo the need for training to support TMH providers in serving culturally diverse patient caseloads, especially those residing in medically underserved communities who are disproportionately at-risk for mental health concerns (31).

Although not specific to cultural competence, TMH providers reported being trained and feeling supported by their professional organization in using telemedicine to practice their specialty. Providers who felt supported in using telemedicine were more likely to have started using telemedicine before the pandemic rather than during it. Weaker perceptions of support among novice telemedicine users may be due to the abrupt, and sometimes mandated shifts from in-person to remote care in March 2020. Harst et al. (22) report that positive attitudes toward telemedicine and its acceptability (e.g., perceived usefulness and ease-of-use) are some of the most important predictors for its personal decision to adopt the technology. However, social policies and organizational infrastructure are also important predictors of telemedicine acceptance, and they are also crucial in considering the long-term adoption and sustainability of telemedicine. In this study, we operationalized facilitating factors as providers’ beliefs about whether they are supported to use telemedicine and adequately trained and provided resources to practice their specialty remotely. Future research is needed to explore the interpersonal, organizational, and policy-oriented factors that facilitate mental health providers’ telemedicine use. Several social and organizational factors have been found to affect providers’ adoption of mobile health solutions in their practice (e.g., workflow, patient, policy/regulation, social influence, monetary factors, evidence-base, awareness, and user engagement) (41). Similar research conducted among TMH providers will begin to inform policy and future procedural practices of telemedicine.

### Limitations

This study was cross-sectional, and it is limited to a single time-point during the COVID-19 pandemic. Surveillance efforts are needed to monitor TMH providers’ perceptions about their delivery of care throughout the remainder of the pandemic and after its resolution. Participant recruitment was limited to users of the Doxy.me telemedicine platform, which may not be representative of all TMH providers or practices. However, participant demographics collected in this study are consistent with those reported in mental health industry statistics (1, 2, 7, 24, 25). Meta-analyses and systematic reviews will be vital to aggregate findings across participant samples and studies. Lastly, these survey data are the product of self-report. Studies in the direct observation of TMH sessions and multicultural care practices will be necessary to understand how providers are adapting to remote care.

### Conclusion

Telemental health providers have positive beliefs about telemedicine one year after the pandemic. They felt satisfied and adequately supported in using telemedicine to provide high-quality care to patients. Providers also reported being capable of supporting a remote, patient-centered environment conducive to openly discussing and evaluating online health resources, cultivating therapeutic alliances, and conducting multicultural competent counseling. However, heterogeneity exists in TMH providers’ perceptions of healthcare delivery according to *when* they adopted telemedicine in relation to COVID-19 and *how much* of their patient caseload is served remotely. Telemedicine is used now more than ever, and providers who hold positive beliefs about the technology are using it with most of their caseload. However, novice TMH providers may require additional training and support to successfully establish a working alliance with their patients, especially those who are multicultural.

## Data Availability Statement

The raw data supporting the conclusions of this article will be made available by the authors, without undue reservation.

## Ethics Statement

The studies involving human participants were reviewed and approved by University of South Florida. The patients/participants provided their written informed consent to participate in this study.

## Author Contributions

All authors contributed equally to conceptualization, research, and manuscript preparation.

## Conflict of Interest

BW is a shareholder, and all other authors are employees of Doxy.me Inc., a commercial telemedicine company.

## Publisher’s Note

All claims expressed in this article are solely those of the authors and do not necessarily represent those of their affiliated organizations, or those of the publisher, the editors and the reviewers. Any product that may be evaluated in this article, or claim that may be made by its manufacturer, is not guaranteed or endorsed by the publisher.

## Abbreviations

COVID-19: Coronavirus Disease 2019
eHEALS: eHealth Literacy Scale
IRB: Institutional Review Board
MCSE-RD: Multicultural Counseling Self-Efficacy Scale – Racial Diversity
MH: Mental Health
TMH: Telemental Health
US: United States
WAI-SR-T: Working Alliance Inventory - Short Revised – Therapist.
